# *Editorial Commentary*: Dead or Alive: Can Viability Staining Predict Response to Tuberculosis Treatment?

**DOI:** 10.1093/cid/ciu1156

**Published:** 2014-12-23

**Authors:** Stephen D. Lawn, Mark P. Nicol

**Affiliations:** 1 Department of Clinical Research, Faculty of Infectious and Tropical Diseases, London School of Hygiene and Tropical Medicine, United Kingdom; 2 Desmond Tutu HIV Centre, Institute for Infectious Disease and Molecular Medicine, Faculty of Health Sciences; 3 Division of Medical Microbiology and Institute for Infectious Diseases and Molecular Medicine, Faculty of Health Sciences, University of Cape Town; 4 National Health Laboratory Service, Groote Schuur Hospital, Cape Town, South Africa

**Keywords:** tuberculosis, viability stain, fluorescein diacetate, multidrug-resistant tuberculosis, treatment response

**(See the Major Article by Datta et al on pages 1186–95.)**

More than 130 years after the discovery of *Mycobacterium tuberculosis*, tuberculosis remains a matter of life or death for millions. In 2013, 9.0 million people worldwide developed the disease and 1.5 million died [1]. The growing problem of multidrug-resistant (MDR) tuberculosis has amplified the toll of this epidemic. An estimated 480 000 new cases of MDR tuberculosis occur annually and, of these, an estimated 9.0% also have extensively drug-resistant (XDR) tuberculosis [1].

The global tuberculosis control strategy aims to achieve rapid diagnosis and effective treatment of tuberculosis, resulting in high cure rates and prevention of onward disease transmission [2]. It is recommended that standardized multidrug treatment regimens are prescribed empirically until the results of drug susceptibility testing (DST) are available [3]. However, the stark reality is that <10% of new tuberculosis cases worldwide had DST done in 2013, reflecting the critical lack of laboratory capacity [1]. More than half of the estimated global burden of MDR tuberculosis cases each year is simply not diagnosed. Blind use of tuberculosis treatment regimens uninformed by DST is undoubtedly a key amplifier of drug resistance.

In the absence of DST results, monitoring patients' response to treatment is especially important. The World Health Organization (WHO) recommends that for patients with pulmonary tuberculosis, sputum smear microscopy for acid-fast bacilli is repeated at the end of the intensive phase of treatment [3]. This, however, is a poor marker of disease activity. Tuberculosis and other mycobacterial infections such as leprosy are characterized by the persistence of nonviable acid-fast bacilli at the site of disease and in clinical samples, regardless of effective treatment [4–6]. Thus, WHO-recommended first-line diagnostic tests for tuberculosis such as sputum smear microscopy and the Xpert MTB/RIF assay, which detects mycobacterial DNA, may remain persistently positive and therefore fail to provide a useful index of disease activity or treatment response [4–6].

Microbiological assessment of the response to tuberculosis treatment requires assays of the viability of *M. tuberculosis* bacilli in clinical samples. As bacterial viability is conventionally defined by the loss of the capacity to divide and form progeny [7], culture is regarded as the reference standard method to determine viability. However, mycobacterial culture requires extended incubation, sophisticated laboratory infrastructure, and highly trained staff. As a result, culture is often not rapid enough to inform clinical decision making and is simply not available in many parts of the world. Various surrogate markers of viability have therefore been used. Viability polymerase chain reaction (PCR) involves use of an agent such as propidium monoazide, which is able to penetrate dead (but not live) bacteria, bind to DNA, and interfere with PCR amplification. Alternatively, reverse transcription PCR can be used to detect short-lived messenger RNA [8] or pre–ribosomal RNA [9], which is synthesized during brief specific nutritional stimulation in vitro. The ability of bacteriophages to enter and multiply within live *M. tuberculosis* has been exploited to determine viability in DST [10]. Importantly, none of these are direct markers of viability, and their kinetics may differ from that of bacterial culture. For example, viability may be rapidly reduced by ultraviolet damage or pasteurization, but cells remain intact and impermeable to propidium iodide or monoazide [7].

In this issue of *Clinical Infectious Diseases*, Datta and colleagues report a clinical evaluation of another viability assay among patients with tuberculosis treated in Peru [11]. This assay does not require sophisticated laboratory infrastructure, and it provides an assessment of mycobacterial viability based on the enzymatic activity of live cells. Esterase activity was measured with a simple staining method using the fatty acid ester fluorescein diacetate. Nonpolar, nonfluorescent fluorescein diacetate enters live bacilli where it is enzymatically hydrolyzed by acetylesterase to polar, fluorescent fluorescein, which rapidly accumulates in the cytoplasm. When viewed under LED (light-emitting diode) fluorescence microscopy, live cells fluoresce green. Dead bacilli lack functional acetylesterase and so do not fluoresce. The latter can be counterstained using ethidium bromide, which readily enters dead cells and intercalates within DNA molecules, whereas functional cell membranes of live bacilli are able to exclude ethidium bromide.

The fluorescein diacetate viability assay was described >30 years ago as a means of assessing the viability of leprosy bacilli, nontuberculous mycobacteria, and *M. tuberculosis* [12, 13]. Field studies have previously evaluated this assay against culture as a means of detecting tuberculosis treatment failure, with variable accuracy being reported [14–17]. Challenges encountered have included limited specificity due to background fluorescence, interfering host tissue debris, and limited sensitivity due to rapid fading of fluorescence [18]. In addition to these few reports where fluorescein diacetate staining was used as a viability assay directly on clinical samples, the technique has also been applied using a rapid flow cytometric read-out when performing DST on clinical culture isolates [19, 20].

In their study, Datta and colleagues studied 35 patients with newly diagnosed smear-positive pulmonary tuberculosis in Peru, among whom most (n = 31) had non-MDR tuberculosis and the remainder (n = 4) had MDR tuberculosis [11]. Treatment response during the first 9 days of first-line therapy was assessed by analyzing sputum samples obtained on days 0, 3, 6, and 9 of therapy. For each patient, the results of fluorescein diacetate viability microscopy were directly compared with those of acid-fast microscopy and with quantitative culture. Whereas the results of acid-fast microscopy altered little during early treatment, a strong relationship was observed between reductions in viability microscopy and reductions in quantitative cultures. Whereas the viability and quantitative culture results approximately halved with each day of treatment in patients with non-MDR tuberculosis, these both remained largely unchanged during treatment of those with MDR tuberculosis. The authors suggest that viability microscopy might therefore be used as an early indicator of poor treatment response and thereby permit early identification of drug-resistant disease. They suggest that viability microscopy may be an appropriate-technology test for use in laboratories in resource-limited settings for this purpose.

The relationship between viability microscopy and quantitative culture demonstrated by Datta and colleagues is strong and plausible. This is the first study to rigorously assess this prospectively over time during early treatment. However, whether this simple assay would be feasible to implement and useful in a programmatic setting remains to be demonstrated. This evaluation represents an important proof-of-concept study, but was limited substantially by the small number of MDR tuberculosis cases. Larger studies are needed to establish the positive and negative predictive values of viability microscopy for MDR tuberculosis.

A number of features of the assay also suggest that it may have limited application in tuberculosis programs. The test is only useful to assess patients with sputum smear–positive pulmonary disease. Because patient assessment with this assay in this study involved comparison of data from samples obtained at >1 time-point during early treatment, this would be challenging to implement programmatically in resource-limited settings. The predictive value of assay results obtained at a single cross-sectional time-point needs to be assessed, as others have done [17]. Although the assay does not require sophisticated laboratory infrastructure or equipment, the required person-time of a laboratory technologist is not an insignificant cost.

A fundamental limitation to this approach is that although viability microscopy may provide early evidence of nonresponse to tuberculosis treatment, it does not identify the underlying cause. Nonresponse may be due to a variety of reasons, including drug resistance, treatment nonadherence, drug malabsorption, and counterfeit drug supplies. Investigation of treatment nonresponse in a patient inevitably requires knowledge of DST results. Arguably, scarce laboratory resources should be prioritized toward establishment of capacity for DST. It is completely unacceptable that in 2013, <10% of the world's tuberculosis cases had DST performed [1]. The advent of rapid molecular assays, such as the Xpert MTB/RIF assay, that are able to detect key drug resistance mutations in the initial diagnostic sample now permit appropriate regimen choices to be made at the start of treatment [21]. The development and implementation of such assays is an important step forward.

Despite skepticism regarding the suggestion that fluorescein diacetate viability microscopy might be widely employed in tuberculosis programs in resource-limited settings as a means of early detection of drug-resistant tuberculosis, the data presented by Datta and colleagues indicate that this simple assay does merit further evaluation. There are a number of additional applications for use in both resource-limited and industrialized settings that would potentially be of value. The relationship between viability microscopy and risk of tuberculosis transmission should be explored. Could this assay be used to define when patients receiving treatment for tuberculosis or MDR tuberculosis are of low infectious risk and no longer require isolation? Establishment of patients with MDR tuberculosis and XDR tuberculosis on effective treatment regimens is challenging. Could fluorescein diacetate viability microscopy be used to assess regimen efficacy in such patients, and could regimens be constructed and modified according to serial assessments of viability microscopy? Similarly, could the assay be used as marker of drug or regimen efficacy in early bactericidal activity studies of new antituberculosis drugs? Further evaluation and development are warranted. This old assay of mycobacterial viability may have life in it yet.
